# Clinical determinants of long-term survival in metastatic uveal melanoma

**DOI:** 10.1007/s00262-021-03090-4

**Published:** 2021-10-28

**Authors:** Elias A. T. Koch, Anne Petzold, Anja Wessely, Edgar Dippel, Michael Erdmann, Lucie Heinzerling, Bettina Hohberger, Harald Knorr, Ulrike Leiter, Friedegund Meier, Peter Mohr, Farnaz Rahimi, Beatrice Schell, Max Schlaak, Patrick Terheyden, Beatrice Schuler-Thurner, Selma Ugurel, Jochen Utikal, Julio Vera, Michael Weichenthal, Fabian Ziller, Carola Berking, Markus V. Heppt

**Affiliations:** 1 Department of Dermatology, Universitätsklinikum Erlangen, Friedrich-Alexander-University Erlangen-Nürnberg (FAU), Erlangen, Germany; 2 Deutsches Zentrum Immuntherapie (DZI), Comprehensive Cancer Center Erlangen-European Metropolitan Area of Nuremberg (CCC ER-EMN), Erlangen, Germany; 3 Department of Dermatology, Ludwigshafen Medical Center, Ludwigshafen, Germany; 4 Department of Dermatology and Allergy, Munich University Hospital (LMU), Munich, Germany; 5 Department of Ophthalmology, Universitätsklinikum Erlangen, Friedrich-Alexander-University Erlangen-Nürnberg (FAU), Erlangen, Germany; 6 Department of Dermatology, Center for Dermatooncology, University Hospital Tübingen, Tübingen, Germany; 7 Department of Dermatology, Skin Cancer Center, Medical Faculty, University Hospital Carl Gustav Carus, TU Dresden, Dresden, Germany; 8 Department of Dermatology, Elbe Klinikum Buxtehude, Buxtehude, Germany; 9 Department of Dermatology, SRH Wald-Klinikum Gera, Gera, Germany; 10 Department of Dermatology, Venerology and Allergology, Charité – Universitätsmedizin Berlin, Corporate Member of Freie Universität Berlin, Humboldt-Universität, Berlin, Germany; 11 Department of Dermatology, Allergology and Venereology, University Medical Center of Schleswig-Holstein, Campus, Lübeck, Germany; 12 Department of Dermatology, University Hospital Essen, University Duisburg-Essen, Duisurg, Germany; 13 Skin Cancer Unit, German Cancer Research Center (DKFZ), Heidelberg, Germany; 14 Department of Dermatology, Venereology and Allergology, University Medical Center Mannheim, Ruprecht-Karl University of Heidelberg, Mannheim, Germany; 15 Department of Dermatology, University Hospital Schleswig-Holstein, Campus Kiel, Kiel, Germany; 16 Department of Dermatology, DRK Krankenhaus Rabenstein, Chemnitz, Germany

**Keywords:** Uveal melanoma, Immune checkpoint blockade, Liver metastases, Liver-directed treatment, Long-term survival, Registry

## Abstract

This study aimed to identify prognostic factors in patients with metastatic uveal melanoma (UM) that were associated with long-term survival in a real-world setting. A total of 94 patients with metastatic UM were included from German skin cancer centers and the German national skin cancer registry (ADOReg). Data were analyzed for the response to treatment, progression-free survival, and overall survival (OS). Prognostic factors were explored with univariate Cox regression, log-rank, and *χ*2-tests. Identified factors were subsequently validated after the population was divided into two cohorts of short-term survival (< 2 years OS, cohort A, *n* = 50) and long-term survival (> 2 years OS, cohort B, *n* = 44). A poor ECOG performance status (hazard ratio [HR] 2.0, 95% confidence interval [CI] 1.0–3.9) and elevated serum LDH (HR 2.0, 95% CI 1.0–3.8) were associated with a poor OS, whereas a good response to immune checkpoint blockade (ICB, *p* < 0.001), radiation therapy (*p* < 0.001), or liver-directed treatments (*p* = 0.01) were associated with a prolonged OS. Long-term survivors (cohort B) showed a higher median number of organs affected by metastasis (*p* < 0.001), while patients with liver metastases only were more common in cohort A (40% vs. 9%; *p* = 0.002). A partial response to ICB was observed in 16% (12/73), being 21% (8/38) for combined ICB, 17% (1/6) for single CTLA4 inhibition, and 10% (3/29) for single PD1 inhibition. One complete response occurred in cohort B with combined ICB. We conclude that the response to ICB and the presence of extrahepatic disease were favorable prognostic factors for long-term survival.

**Supplementary Information:**

The online version contains supplementary material available at 10.1007/s00262-021-03090-4.

## Introduction

Uveal melanoma (UM) is the most common tumor of the eye in adults but still represents a rare subtype of melanoma. The mean age-adjusted incidence is 5.2 per million [1] and shows a north-to-south decreasing gradient in Europe [2], with the highest current mean incidence of 9.5 per million in Ireland [3]. The treatment approach of the primary tumor depends on the tumor size, patient preference, and tumor localization, most commonly by brachytherapy or enucleation [4, 5]. The most important risk factor for the development of metastases is monosomy 3, and at least 40–50% of patients will develop metastases, predominantly to the liver [6, 7]. Since there is no standardized and effective treatment for advanced UM, the prognosis is bleak once metastases develop [8]. A meta-analysis of studies published between 1980 and 2017 including 2494 patients calculated a median overall survival (OS) across all treatment modalities of 1.07 years [9]. However, the population of this meta-analysis was treated mainly in the time before immune checkpoint blockade (ICB) was available. In both settings, some patients show a more favorable disease course with longer OS. Thus, this study aimed to identify parameters that are linked to a more favorable clinical outcome in patients with metastatic UM.

## Patients and methods

### Patient population and data sources

We performed a retrospective, multi-center, explorative analysis. Patients with metastatic UM receiving any treatment regime were eligible. Inclusion criteria were histologically confirmed stage IV UM and a follow-up time of at least three months. As we were interested in determinants of long-term survival, the following exclusion criteria applied: (1) unknown survival status within two years after diagnosis of stage IV disease, (2) unknown date of entry into stage IV, and (3) ongoing treatment and a survival time of fewer than two years at the data cut-off. These criteria were set as it was unclear at the time of the data cut-off if patients under these conditions would, later on, turn out as long-term survivors. Clinical data and the treatment outcomes of interest were extracted from the original patient records from three German skin cancer centers (Universitätsklinikum Erlangen *n* = 43, University Hospital Munich *n* = 3, and Klinikum Ludwigshafen *n* = 2), as well as from the ADOREG registry of the German Dermatologic Cooperative Oncology Group (DeCOG, *n* = 46). Data were collected and merged into a central database before analysis. The ADOReg registry is a large prospective clinical database in the field of dermatologic oncology collecting data to generate high-quality real-world evidence. This study was approved by the scientific board of the registry and by the institutional review board of the medical faculty of the Munich University Hospital (approval number 413–16 UE). Furthermore, it was conducted following the principles of the Helsinki Declaration in its current version.

### Data collection and treatment outcomes

The clinical data recorded at the diagnosis of stage IV UM (“baseline”) comprised demographics such as sex, age, number, and sites of metastases, the Eastern Cooperative Oncology Group (ECOG) performance status, and serum lactate dehydrogenase (LDH) as a dichotomous variable (elevated vs. not elevated; cut-off 250 U/l). The most common metastatic sites such as liver, bone, lung, and central nervous system (CNS) were specified, while all other localizations were summarized within the category “other metastases.” No further information was available on the extent of hepatic metastasis.

We recorded the number and types of treatments and further dissected ICB regarding start and end date, time to progression, and best radiologic response evaluation based on the RECIST criteria version 1.1. The best radiologic response to ICB treatment which was achieved during the disease course was assessed by the site investigators and indicated as complete response (CR), partial response (PR), stable disease (SD), or progressive disease (PD). In all cases, patients were treated until disease progression or development of unacceptable toxicity. As the treatments were highly heterogeneous, we subsumed interventions other than ICB, cytotoxic chemotherapy, and vaccination with dendritic cells loaded with tumor-intrinsic RNA (DC) as a category “other treatments.” Besides, we extracted data on the performance of liver-directed treatments and radiation therapy.

### Statistical analysis

OS was calculated as the time from the diagnosis of stage IV UM until melanoma-specific or treatment-related death. The progression-free survival (PFS) was determined as the time from treatment start until disease progression. Time-to-event analyses were calculated where death or progression was considered as an event. If neither occurred or if patients were lost to follow-up, the date of the last documented presentation was used as a censored observation.

The survival and progression probabilities were estimated with the Kaplan–Meier method. To test for a significant moderating factor, the survival curves were compared with the log-rank test. The hazard ratio (HR) with 95% confidence interval (CI) was calculated by the COX proportional hazard regression whenever the ability for assuming the proportional hazards was given (no crossing of survival curves in the log–log plot). In this case, the *p*-values were calculated with the Wald test. If the proportional hazard assumption was violated, only the different median OS was indicated. Patients with missing values for a given variable were excluded. No imputation of missing data was performed.

For investigating possible factors being significantly different in the groups of short-time survivors (cohort A) vs. long-time survivors (cohort B), log-rank tests, *χ*^2^–tests and t-tests were performed. In all cases, two-tailed p-values were calculated and considered significant with values *p* < 0.05. All analyses were carried out with the software R (https://www.r-project.org/) using the packages “survival” and “survminer.”

## Results

### Characteristics of the study population

A total of 94 (100%) patients with metastatic UM met the eligibility criteria and were included (Table 1). A median of three organ systems was affected by metastases, predominantly liver (93%), lung (51%), and bones (34%); 42% had an ECOG performance status of 0. Serum LDH was elevated in 38% when stage IV disease was entered.

**Table 1:** Characteristics of the study population

Parameter	Categories	Number (%) *N* = 94 (100%)	Group A *N* = 50	Group B *N* = 44	*p*-value
Age	Median in years	67 (range 33–92)	67.4	65.8	*p* = 0.85
Sex	Female	41 (44%)	22 (44%)	19 (43%)	*p* = 1
	Male	53 (55%)	28 (56%)	25 (57%)	
ECOG performance status	0	39 (42%)	17 (34%)	22 (50%)	*p* = 0.17
	1	12 (13%)	5 (10%)	7 (16%)	
	2	3 (3%)	3 (6%)	0 (0%)	
	3	1 (1%)	1 (2%)	0 (0%)	
	4	0 (0%)	0 (0.0%)	0 (0.0%)	
	Not indicated	39 (42%)	24 (48%)	15 (34%)	
LDH	Not elevated	22 (23%)	9 (18%)	13 (30%)	*p* = 0.02
	Elevated	36 (38%)	27 (54%)	9 (20%)	
	Not indicated	36 (38%)	14 (28%)	22 (50%)	
Sites of metastasis	Liver	87 (93%)	48 (96%)	39 (89%)	*p* = 0.3
	Hepatic only	24 (26%)	20 (40%)	4 (9%)	*p* = 0.001
	Both hepatic and extrahepatic	63 (67%)	28 (56%)	35 (80%)	*p* = 0.028
	Extrahepatic only	7 (7%)	2 (4%)	5 (11%)	*p* = 0.34
	Pulmonary	48 (51%)	20 (40%)	28 (64%)	*p* = 0.04
	Bone	32 (34%)	16 (32%)	16 (36%)	*p* = 0.8
	CNS	20 (21%)	7 (14%)	13 (30%)	p = 0.1
	Other sites	42 (45%)	13 (26%)	29 (66%)	*p* < 0.001
	Not indicated	2 (2%)	0 (0%)	2 (5%)	*p* = 0.42
Number of metastatic sites	Median (range)	3 (1–5)	2 ( 1–5)	3 (1–5)	*p* < 0.001
Systemic treatments	Vaccination with dendritic cells	6 (6.4%)	1 (2.0%)	5 (11.4%)	*p* = 0.15
	Any immune checkpoint blockade	81 (86%)	41 (82%)	40 (91%)	*p* = 0.15
	Single CTLA4 inhibition	8 (9%)	2 (4%)	6 (14%)	*p* = 0.19
	Single PD-1 inhibition	33 (35%)	17 (34%)	16 (38%)	*p* = 0.98
	Combined immune checkpoint blockade	40 (43%)	22 (44%)	18 (41%)	*p* = 0.93
	Not indicated	2 (2%)	1 (2%)	1 (2%)	*p* = 1
	Chemotherapy	29 (31%)	9 (18%)	20 (46%)	*p* = 0.02
	Reinduction with immune checkpoint blockade	33 (35%)	9 (18%)	24 (55%)	*p* = 0.001
Liver-directed treatment	Yes	35 (37%)	17 (34%)	18 (41%)	*p* = 0.01
	No	41 (44%)	32 (64%)	9 (21%)	
	Unknown	18 (19%)	1 (2%)	17 (39%)	
Radiation therapy	Yes	23 (25%)	8 (16%)	15 (34%)	*p* = 0.001
	No	53 (56%)	42 (84%)	9 (21%)	
	Unknown	18 (19%)	0 (0.%)	18 (41%)	

**Fig. 1:**
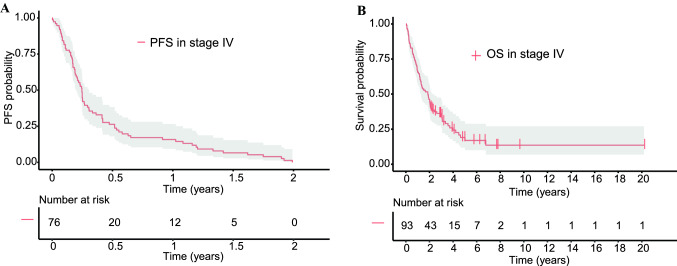
Kaplan–Meier estimates of the patient population for (**A**) progression-free survival (PFS) to first systemic therapy and (**B**) overall survival (OS). The median PFS and OS were 3.0 months (95% CI 2.4–3.7) and 22.3 months (95% CI 14.9–26.7), respectively

The median OS of the entire population was 22.3 months (95% CI 14.9–26.7), the median PFS in stage IV disease after the first systemic treatment was 3.0 months (95% CI 2.4–3.7) (Fig. 1A + B). The majority of patients received any ICB (86%), while liver-directed treatments and radiation therapy were applied in 37% and 25%, respectively. One CR occurred in a patient undergoing combined ICB (1%, 1/73). The PR rate for all ICB regimens was 16% (12/73), being 21% for combined ICB (8/38), 17% for single CTLA4 inhibition (1/6), and 10% for single PD1 inhibition (3/29, Table 2). The median PFS after ICB was 2.5 months (95% CI 2.1–3.5). In cohort B, the rate of PR was 14% (2/14) and 41% (7/17) for single PD1 inhibition and combined ICB, respectively. One CR (6%, 1/17) occurred in this cohort (Table 3).

**Table 2:** Partial response rates of short-term (cohort A) versus long-term (cohort B) survivors

ICB regimen	Total (*n*)	Cohort A (*n*)	Cohort B (*n*)	*p*-value
Any ICB	16% (12/73)	5% (2/38)	29% (10/35)	*p* = 0.018
Single PD1 inhibition	10% (3/29)	7% (1/15)	14% (2/14)	*p* = 0.95
Single CTLA4 inhibition	17% (1/6)	0% (0/2)	25% (1/4)	*p* = 1
Combined ICB	21% (8/38)	5% (1/21)	41% (7/17)	*p *= 0.019

*ICB* immune checkpoint blockade

**Table 3:** Response rates to ICB in long-term survivors (cohort B)

ICB regimen (cohort B)	Complete response	Partial response	No response (stable disease, progressive disease)
Any ICB (*n* = 35)	1 (3%)	10 (29%)	24 (69%)
Single PD1 inhibition (*n* = 14)	0 (0%)	2 (14%)	12 (86%)
Single CTLA4 inhibition (*n* = 4)	0 (0%)	1 (25%)	3 (75%)
Combined ICB (*n* = 17)	1 (6%)	7 (41%)	9 (53%)

*ICB* immune checkpoint blockade

### Identification of prognostic factors

**Fig. 2:**
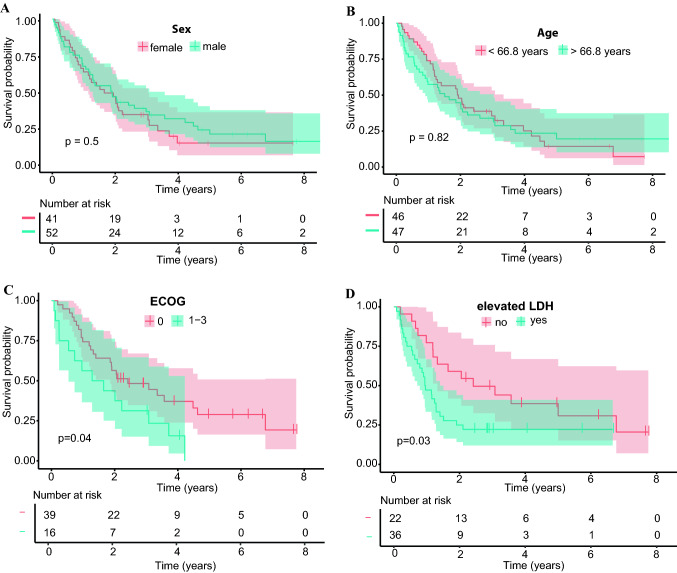
**A** Kaplan–Meier estimates for overall survival (OS) according to sex. The median OS was 19.7 (95% CI 13.8–36.4) for females vs. 22.6 months (95% CI 14.2–37.4) for males. **B** OS according to age. The median OS was 23 (95% CI 14.8–40.1) for < 66.8 years vs. 18.2 months (95% CI 10.9–36.4) for > 66.8 years. **C** OS according to ECOG performance status (HR≈2, *p* = 0.04). **D** OS according to serum LDH level (HR≈2, *p* = 0.03)

We next performed univariate analysis on the entire population to identify putative prognostic factors. Neither sex nor age was associated with OS (Fig. 2A + B). Both a poor ECOG performance status (HR 2.0, 95% CI 1.0–3.9) and elevated serum LDH level (> 250 U/l; HR 2.0, 95% CI 1.0–3.8) were unfavorable prognostic factors (Fig. 2C + D). The site and number of metastases were not associated with OS except for the category “other metastases” (*p* = 0.009) (Suppl. Figure 1, Suppl. Table 1). Patients with liver metastases only showed significantly worse survival compared to those with a combination of both hepatic and extrahepatic metastases (median OS 7.7 vs. 24.8 months, respectively; *p* = 0.019; Suppl. Figure 1 G).

**Fig. 3:**
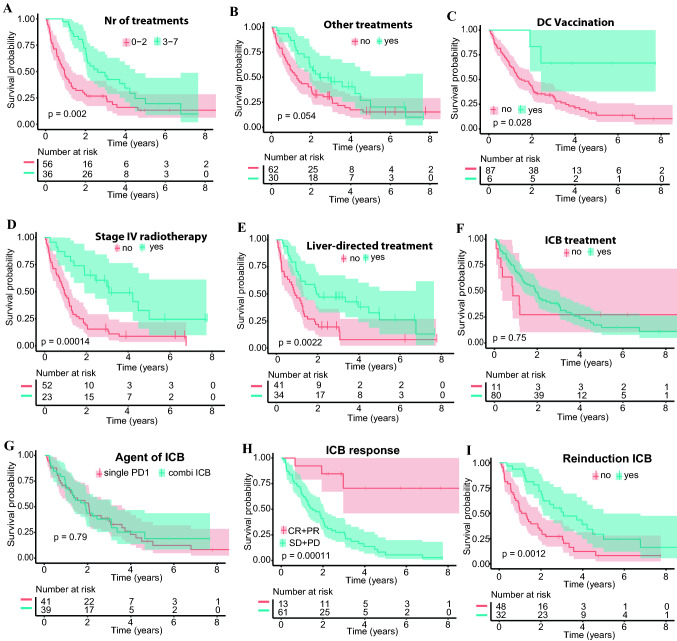
**A** Kaplan–Meier estimates for overall survival (OS) according to the number of treatments. The median OS was 11.3 (95% CI 7.9–16.3) for patients with ≤ 2 treatment lines vs. 31.3 months (95% CI 24.7–53.8) for those with ≥ 3 treatment lines. **B** OS according to “other” therapies. The median OS was 15.5 (95% CI 14.6–24.5) for patients with no “other” therapies vs. 29.0 months (95% CI 22.9-NR with “other” therapies. **C** OS according to DC vaccination (HR≈0.24, *p* = 0.04). The median OS was 18.2 months (95% CI 14.2–24.8) for patients without DC vaccination and not reached with DC vaccination. **D** OS according to radiation therapy in stage IV (H≈0.33, *p* < 0.001). The median OS was 11.3 (95% CI 8.6–15.5) for patients without radiation vs. 27.0 months (95% CI 22.9-NR) for those receiving radiation. **E** OS according to liver-directed treatments (HR≈0.44, *p* = 0.003). The median OS was 10.9 (95% CI 7.6–15.9) for patients without liver-directed treatments vs. 24.0 months (95% CI 14.2-NR) with liver-directed treatments. **F** OS according to immune checkpoint inhibitor blockade (ICB). The median OS was 10.3 (95% CI 4.5-NR) vs. 23.1 months (95% CI 16.3–35.8). **G** OS according to different agents of ICB. The median OS was 24.5 (95% CI 15.4–42.9) for patients with single ICB treatment vs. 22.8 months (95% CI 15.5–40.2) with combined ICB. **H** OS according to the response to ICB. The median OS was not reached for patients with complete or partial response (CR + PR) vs. 18.2 months (95% CI 13.8–24.8) for those without a response. **I** OS according to ICB reinduction (HR≈0.48, *p* = 0.004). The median OS was 14.1 (95% CI 10.9–23.3) for patients without ICB reinduction vs. 37.0 months (95% CI 25.3–60.1) with reinduction. NR = not reached

Regarding treatments, the univariate analysis demonstrated prolonged survival of patients with three or more treatment lines (*p* = 0.002), “other treatments” (*p* = 0.054), and DC vaccination although the absolute numbers were low (HR 0.24, 95% CI 0.06–0.96; Fig. 3A–C, Suppl. Table 4). Also, patients undergoing radiation therapy (HR 0.33, 95% CI 0.18–0.60) or liver-directed treatments (HR 0.44, 95% CI 0.26–0.76) showed more favorable OS (Fig. 3D + E, Suppl. Table 4). The best response to ICB was a strong prognostic factor (*p* < 0.001; Fig. 3H), as was the reinduction of ICB (*p* = 0.001; Fig. 3I, Suppl. Table 4). There was no significant difference in OS between single PD1 and combined ICB (*p* = 0.79; Fig. 3G, Table Suppl. Table 4).

### Comparison of short-term and long-term survivors

**Fig. 4:**
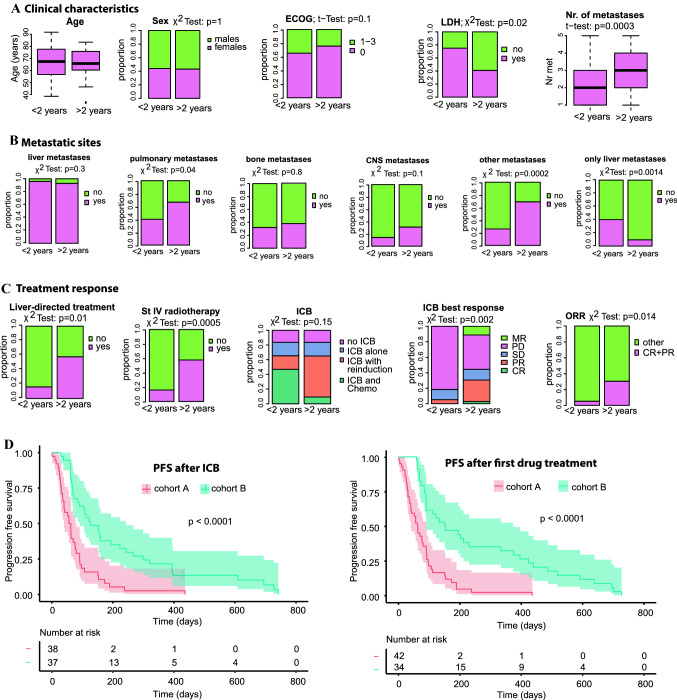
Investigation of the distribution of prognostic factors in short-term (cohort A) vs. long-term (cohort B) survivors. **A** Clinical characteristics; **B** metastatic sites; **C** patterns of treatment response. **D** Kaplan–Meier estimates comparing the cohorts A and B for progression-free survival (PFS) after immune checkpoint inhibitor blockade (ICB) and after any first drug treatment in stage IV. The median PFS in stage IV disease after ICB was 1.9 (95% CI 1.3–2.6) for cohort A vs. 4.2 months (95% CI 3.0–8.0) for cohort B (left). After the first drug therapy, the median PFS was 2.1 (95% CI 1.3–3.0) for cohort A vs. 5.0 months (95% CI 3.0–12.3) for cohort B (right)

To further validate prognostic factors regarding long-term OS, the study population was subsequently divided into two subgroups: (1) cohort A (*n* = 50) with patients who died within the first two years after they entered stage IV disease and (2) cohort B (*n* = 44) consisting of patients with a survival of longer than 2 years. Age, sex, and ECOG performance status were evenly distributed among both cohorts (Fig. 4A). In contrast, elevated serum LDH levels were more commonly observed in cohort A (*p* = 0.02). Interestingly, patients in cohort B had a higher median number of affected organ systems (*p* < 0.001), including more patients with lung (*p* = 0.04) and “other metastases” (*p* < 0.01), while liver, bone, and CNS metastases were evenly distributed (Fig. 4B). Patients with liver metastases only were more common in cohort A than in B (40% vs. 9%; *p* = 0.002).

Both cohorts were treated equally often with ICB, but the frequency of achieving a partial response was significantly higher in cohort B (29%) than in A (5%; *p* = 0.018) (Table 2). Strikingly, the PR to combined ICB was 5% vs. 41% for cohorts A and B, respectively (*p* = 0.019). The median PFS after ICB was 1.9 months (95% CI 1.3–2.6) vs. 4.2 months (95% CI 3.0–8.0) for cohorts A and B, respectively (*p* < 0.001; Fig. 4D). ICB reinduction was more frequent in cohort B (*p* = 0.001). Also, long-term survivors more commonly underwent radiation therapy (*p* < 0.001) or liver-directed treatments (*p* = 0.01; Table 1). The median PFS to the first palliative treatment of any type was 1.9 months (95% CI 1.9–3.1) vs. 5.0 months (95% CI 2.7–13.6), respectively (*p* < 0.001; Fig. 4D).

## Discussion

Considering that there are no standardized treatment options and only a few high-quality trials for metastatic UM, data from a real-world setting and registries are of particular value for this orphan cancer condition. In this study, we followed a two-step strategy to identify clinical parameters that are associated with a favorable outcome. First, we assembled a population from multiple centers within Germany and registry data and performed univariate analyses to explore possible prognostic factors. Second, we further validated these parameters after the population was divided into two cohorts with survival of less and more than 2 years as a proxy for short-term and long-term survival, respectively. Thus, patients with an unknown death status or ongoing treatment within two years after entry into stage IV were excluded. This selection of the population may explain why the OS was relatively high compared to previous studies [10–12]. The median OS for the entire population was 22.3 months which is by far longer than the proposed benchmark of 10.2 months in a recent meta-analysis of 912 patients from clinical trials but comparable to monocentric data other sites [13, 14]. Furthermore, this difference could be explained by a higher proportion of patients treated with ICB. The benchmark study included patients from numerous clinical trials before ICB was available. Accordingly, the proportion of patients receiving immunotherapy was also only 15%, while 34% were still treated with chemotherapy [14]. In our study, a large proportion received ICB at least once in stage IV disease. The PR rate to single PD1 inhibition and combined ICB were 10% and 21%, respectively. These results are slightly higher than in two recently published phase II trials [15, 16]. Piulats et al. achieved a PR rate of 10% in 52 treatment-naïve patients in the Spanish GEM-1402 trial. A higher PR rate of 15% was reported by Pelster et al. in 33 patients with any prior treatment for UM. One CR occurred in each of these two prospective studies. Similarly, Pelster et al. reported more favorable OS and PFS values than in the Spanish trial (5.5 vs. 3 months and 19.1 vs. 12.7 months, respectively). These data are in line with our results from a real-world setting and clearly beat the benchmark values [17]. Although the response rates to ICB are by far lower than in cutaneous melanoma, we conclude that the overall prognosis of metastatic UM has been improved significantly by the introduction of ICB.

Our results confirm that a good ECOG performance status and normal serum LDH levels are important prognostic factors in stage IV UM, as was outlined by us and others [11] [18]. Male sex and advanced age were not correlated with survival in contrast to the benchmark data. Interestingly, the number of affected organs was significantly higher in cohort B, while patients with liver metastases only were more commonly observed in cohort A. Also, patients with liver metastases only showed significantly worse survival compared to those with a combination of both hepatic and extrahepatic metastases. This distribution is intriguing and was also observed in the two recent phase II studies. Piulats et al. reported that the OS in patients with exclusive liver metastases was shorter than that in patients with metastases in other locations beyond the liver (9.2 vs. 23.5 months) and those with both liver and other metastases (15.5 months) [15, 16]. Pelster et al. achieved a response in 6 patients of that 5 had both liver and extrahepatic metastases. These results imply that exclusive hepatic metastases are a major unfavorable prognostic factor. The liver exerts a particularly immunosuppressive effect, and the hepatic tumor microenvironment may prevent antigen recognition by tumor-infiltrating lymphocytes, even when treated with ICB [19]. However, if extrahepatic metastases are present, this immunosuppressive effect can be bypassed and the resistance to ICB can be overcome more easily. This hypothesis can help to explain the feeble immune response in UM as 95% of the metastases spread to the liver which is, in general, the least responsive metastatic site to ICB [6, 20]. Also, 93% of the patients included in our study had liver metastases with no significant difference between the cohorts (96% vs 89%), whereas the long-term survivors had significantly more frequently pulmonary and “other” metastases. Besides, the number of patients in cohort B with exclusive liver metastases was substantially lower (20 vs. 5 patients). It is noteworthy that the patients with liver metastases only had shorter OS compared to those with several metastatic sites (7.7 vs. 24.8 months) although the overall tumor burden is likely to be higher in the latter group. Consequently, rapidly progressive liver metastases were, counterintuitively, associated with a worse overall survival, while patients with extrahepatic metastasis had a more favorable disease course. We conclude that the presence of multiple affected organ systems with extrahepatic disease is associated with either a more indolent disease course per se or results in better treatment responses. These data support another interesting hypothesis, i.e., that liver metastases cause the elimination of CD8^+^ T-cells via immunosuppressive effects of macrophages [21]. This elimination of T-cells may lead to a poor response to ICB in patients with liver metastases only. Besides, liver metastases of UM have a significantly lower PD-L1 expression compared to metastases of cutaneous melanoma, which might also be a reason for the poor response [22–24]. Interestingly, the absence of melanin in liver metastases of UM induces stronger immune responses and is associated with an improved ICB response [25]. However, the hepatic tumor microenvironment might be influenced positively by liver-directed treatments. In our population, patients receiving liver-directed treatments showed significant survival advantages. It remains unclear whether this benefit is truly achieved by liver-directed treatment or whether patients with a more isolated disease are selected for this procedure that *per se* have a better prognosis. Nonetheless, these interesting signals observed by us and others should be further investigated in future studies.

The limitations of our study are its retrospective design and the exclusion of patients with an unknown death status within 2 years after entry into stage IV that may positively bias the survival. Also, the genotype has proven influence on the prognosis but was not considered here due to missing data as no data on the genetic background of the tumors were available.

To date, we have some information on the impact of genetic alterations (1) on development and the pattern of metastasis formation, (2) on the disease course once metastasis has developed, and (3) on the treatment response, in particular to ICB. UM is genetically characterized by chromosomal losses and gains as well as a low mutational burden. Chromosomal alterations like monosomy 3, disomy 3, and gain of chromosome 8q are considered strong prognostic factors. Monosomy 3 is associated with poor disease-free survival (DFS), while tumors with disomy 3 tend to spread rarely and have prolonged DFS. Gain of chromosome 8q and loss of chromosome 3 are associated with a poor OS [26, 27]. Somatic GNAQ and GNA11 mutations are commonly detected leading to consecutive activation of the MAPK pathway which might contribute to the development of UM [28]. However, survival seems not to be affected by these mutations [29]. Further, hemizygous mutations in the BAP-1 gene were found in monosomy 3 tumors resulting in loss or dysfunctional BAP-1 expression associated with poor DFS and OS [30–32]. Szalai et al. demonstrated a strong association between BAP-1 mutated tumors and a peak of clinical detected metastases 2 years after enucleation [33]. They suggested that this might be an explanation for the observations of the disproved Zimmerman–McLean–Forster effect, who hypothesized that the enucleation causes a spread of tumor cells leading to the peak of metastases [33, 34]. However, BAP-1 mutated tumors might correlate to locally fast progressive liver metastasis observed in our study population. Moreover, SF3B1 and EIF1AX mutations occur in tumors with disomy 3 and are associated with a more favorable disease course [32, 35, 36]. SF3B1 mutations correlate with a long DFS and with a late peak of metastases after 7 years [30, 33]. EIF1AX mutations as well as wild-type BAP-1, SF3B1, and EIF1AX genes are correlated with prolonged survival [30]. Thus, the genotype plays an important role in determining the overall prognosis regardless of treatments. Further, there are signals that a high mutational burden is predictive for response to ICB [37] and a MBD4-related hypermutator phenotype in metastatic uveal melanoma showed an exceptional high sensitivity to anti-PD-1 which is present in up to 2% of UM patients [38].

Altogether, the prognosis of metastatic UM in this population was better than the recently proposed benchmark and may have improved by the more frequent use of ICB. We demonstrate that a good response to ICB, extrahepatic disease, and liver-directed treatments, were associated with long-term survival.

### Supplementary Information

Below is the link to the electronic supplementary material.

Supplementary file1 (AI 921 KB)

Supplementary file2 (DOCX 14 KB)

Supplementary file3 (DOCX 14 KB)
