# Supplementary material for: Clinical determinants of long-term survival in metastatic uveal melanoma
Source: Cancer Immunol Immunother. 2021 Oct 28;71(6):1467–77. doi: 10.1007/s00262-021-03090-4 (PMC9123041; doi:10.1007/s00262-021-03090-4)
Supplement: Supplementary file 2 — Supplementary file2 (DOCX 14 KB) [file 262_2021_3090_MOESM2_ESM.docx]

**Supplementary Table 1: Overall survival according to metastatic sites.**

| **Parameter** | **Category** | **Median OS in months** | **95% confidence interval in months** | **p-value of the log-rank test** |
| --- | --- | --- | --- | --- |
| Number of metastases | 1-2 | 14.3 | 8.6-23.3 | p=0.2 |
|  | 3-5 | 25.1 | 22.3-44.6 |  |
| Liver metastases | No | NR | NR | p=0.35 |
|  | Yes | 21.1 | 14.3-26.7 |  |
| Bone metastases | No | 19.8 | 14.3-37.0 | p=0.24 |
|  | Yes | 23.2 | 11.3-37.0 |  |
| Other metastases | No | 11.4 | 7.6-16.9 | p=0.009 |
|  | Yes | 35.8 | 25.3-50.7 |  |
| Pulmonary metastases | No | 15.5 | 11.5-23.3 | p=0.42 |
|  | Yes | 24.8 | 16.9-44.6 |  |
| CNS metastases | No | 15.5 | 12.8-24.7 | p=0.17 |
|  | Yes | 27.2 | 22.9-NR |  |
| Liver metastases only | No | 24.8 | 22.3-37.0 | p=0.019 |
|  | Yes | 7.7 | 4.5-15.9 |  |

Abbreviations: OS=overall survival, NR=not reached, CNS=central nervous system.
