# Supplementary material for: Clinical determinants of long-term survival in metastatic uveal melanoma
Source: Cancer Immunol Immunother. 2021 Oct 28;71(6):1467–77. doi: 10.1007/s00262-021-03090-4 (PMC9123041; doi:10.1007/s00262-021-03090-4)
Supplement: Supplementary file 3 — Supplementary file3 (DOCX 14 KB) [file 262_2021_3090_MOESM3_ESM.docx]

**Table 4: Overall survival according to treatment modalities.**

| **Parameter** | **Category** | **Number of patients** | **Median OS in months** | **95% confidence interval in months** | **p-value of the log-rank test** |
| --- | --- | --- | --- | --- | --- |
| Number of treatments | 0-2 | 56 | 11.3 | 7.9-16.3 | p=0.002 |
|  | 3-7 | 36 | 31.3 | 24.7-53.8 |  |
| “Other” therapies | No | 62 | 15.5 | 14.6-24.5 | p=0.054 |
|  | Yes | 30 | 29.0 | 22.9-NR |  |
| DC vaccination | No | 87 | 18.2 | 14.2-24.8 | p=0.03 |
|  | Yes | 6 | NR | NR |  |
| Stage IV radiation therapy | No | 52 | 11.3 | 8.6-15.5 | p<0.001 |
|  | Yes | 23 | 27.0 | 22.9-NR |  |
| Liver-directred treatment | No | 41 | 10.9 | 7.6-15.9 | p=0.002 |
|  | Yes | 34 | 24.0 | 14.2-NR |  |
| ICB treatment | No | 11 | 10.3 | 4.5-NR | p=0.75 |
|  | Yes | 80 | 23.1 | 16.3-35.8 |  |
| Agent of ICB | single | 41 | 24.5 | 15.4-42.9 | p=0.79 |
|  | combined | 39 | 22.8 | 15.5-40.2 |  |
| ICB response | CR+PR | 13 | NR | NR | p<0.001 |
|  | SD+PD | 61 | 18.2 | 13.8-24.8 |  |
| Reinduction ICB | No | 60 | 14.1 | 10.9-23.3 | p=0.001 |
|  | Yes | 33 | 37.0 | 25.3-60.1 |  |

Abbreviations: OS=overall survival, ICB=immune checkpoint blockade, NR=not reached, CR+PR=complete and partial response, SD+PD=stable and progressive disease.
